# Effects of electrotactic exercise and antioxidant EUK-134 on oxidative stress relief in *Caenorhabditis elegans*

**DOI:** 10.1371/journal.pone.0245474

**Published:** 2021-01-20

**Authors:** Thi Thanh Huong Pham, Wan-Ying Huang, Chang-Shi Chen, Wen-Tai Chiu, Han-Sheng Chuang

**Affiliations:** 1 Department of Biomedical Engineering, National Cheng Kung University, Tainan City, Taiwan; 2 Department of Biochemistry and Molecular Biology, National Cheng Kung University, Tainan City, Taiwan; 3 Center for Micro/Nano Science and Technology, National Cheng Kung University, Tainan City, Taiwan; Sri Ramachandra Institute of Higher Education and Research, Chennai, India, INDIA

## Abstract

Antioxidant uptake and regular exercise are two well-acknowledged measures used for rejuvenation and oxidative stress elimination. Previous studies have revealed that moderate exercise mildly increases intracellular signaling oxidant levels and strengthens the ability of an organism to deal with escalating oxidative stress by upregulating antioxidant enzymes, such as superoxide dismutase (SOD), catalase (CAT), and glutathione peroxidase. Antioxidant supplementation directly scavenges intracellular reactive oxygen species (ROS) to reduce oxidative stress. However, research to understand the impacts of these enzymes on mitigating oxidative stress from the perspective of simple animals is limited. Herein, we show that exercise combined with antioxidant supplementation ameliorates the physiological phenotypes and markers of aging in wild-type and SOD/CAT-deficient *Caenorhabditis elegans*. We discovered that treated wild-type and gene-deficient worms show better survivorship, reproduction, and motility compared with their control counterparts. Assays of biochemical indices revealed that variations in *sod-3* expression under different stress levels imply an inducible enzyme response resulting from exercise training and antioxidant supplementation. In addition, induced ROS resistance obtained from any type of treatment could persist for several days even after treatment cessation, thus suggesting a potential long-term antioxidative stress effect. Our findings confirm that exercise, antioxidant supplementation, and their combination could significantly improve the ability of *C*. *elegans* to withstand adverse stress. Our observations provide promising insights into future therapies of anti-oxidative stress in higher animals.

## Introduction

Daily life triggers the production of various reactive oxygen species (ROS) originating from exogenous and endogenous sources. ROS are a major byproduct of metabolic reactions generated by the mitochondria. At low levels, these species are involved in various biological processes as signaling molecules [1–3] and immune barriers and combat bacterial invasion [4]. However, ROS accumulation can pose a threat to human health. Oxidative stress resulting from free radical attack may cause cell damage and pathological conditions, including age-related diseases [5, 6]. The oxidative stress model is often used to explain how ROS disturbs cellular functions by impairing DNA or proteins [7, 8]. Besides metabolic ROS, human bodies are constantly exposed to various oxidative stressors, such as air pollution, heavy metals in water, and food poisoning [9]. Thus, scavenging of excess ROS is essential to maintain homeostasis in daily physiological functions. Mild ROS induced by physical training can strengthen the antioxidant capacity of cells by triggering stimuli in endogenous antioxidant pathways [10, 11]. Mild and regular exercise has been proven to be a beneficial lifestyle intervention to improve fitness during the healthy state and enable the early diagnosis of numerous diseases [12]. However, strenuous exercise produces excess ROS, which can impair intracellular enzymes and organelles and activate programmed cell death [13–18]. Another exogenous method to mitigate ROS damage is antioxidant supplementation [19]. Intake of external non-enzymatic antioxidants improves cell damage by neutralizing ROS [20, 21]. Prior studies have proven that appropriate intake of external non-enzymatic antioxidants, for instance, vitamins C and E, helps scavenge harmful ROS to reduce accumulation of oxidative stress in cells [22–25]. Although both endogenous and exogenous measures lead to the same consequence of oxidative stress relief, the anti-oxidative effect of a treatment combining both exercise and antioxidant supplementation on *Caenorhabditis elegans* is yet unknown.

Considering its simplicity, *C*. *elegans* is a well-known model that can provide valuable clues to the genetic and molecular mechanisms underlying the intricacies of human diseases and aging. In our past study [13, 26, 27], we introduced an electrotactic microchip to control *C*. *elegans* under exercise via their electrotactic nature [28, 29]. Notably, in comparison to the randomly swim exercise reported by Laranjeiro et al. [30], our actively-controlled method created “forced movement”. Therefore, consistent force and timing was achievable. Our findings revealed that ROS levels in worms of all ages escalate shortly after exercise training but decrease to low levels 6 h later. Other studies have also reported that supplementation with antioxidant compounds, such as vitamins C and E, can extend lifespans and reduce oxidative stress [31–33]. However, critical evidence supporting the benefits of antioxidant supplementation to exercise performance and recovery remains lacking [34]. In the current study, we employ the synthetic superoxide dismutase/catalase (SOD/CAT) mimetic EUK-134 as an extracellular antioxidant source and examine its synergistic effect with exercise on ROS relief. This antioxidant can function as supplemental SOD/CAT to neutralize O_2_^−^ and H_2_O_2_ and reduce oxidative stress in worms [35].

**Fig 1:**
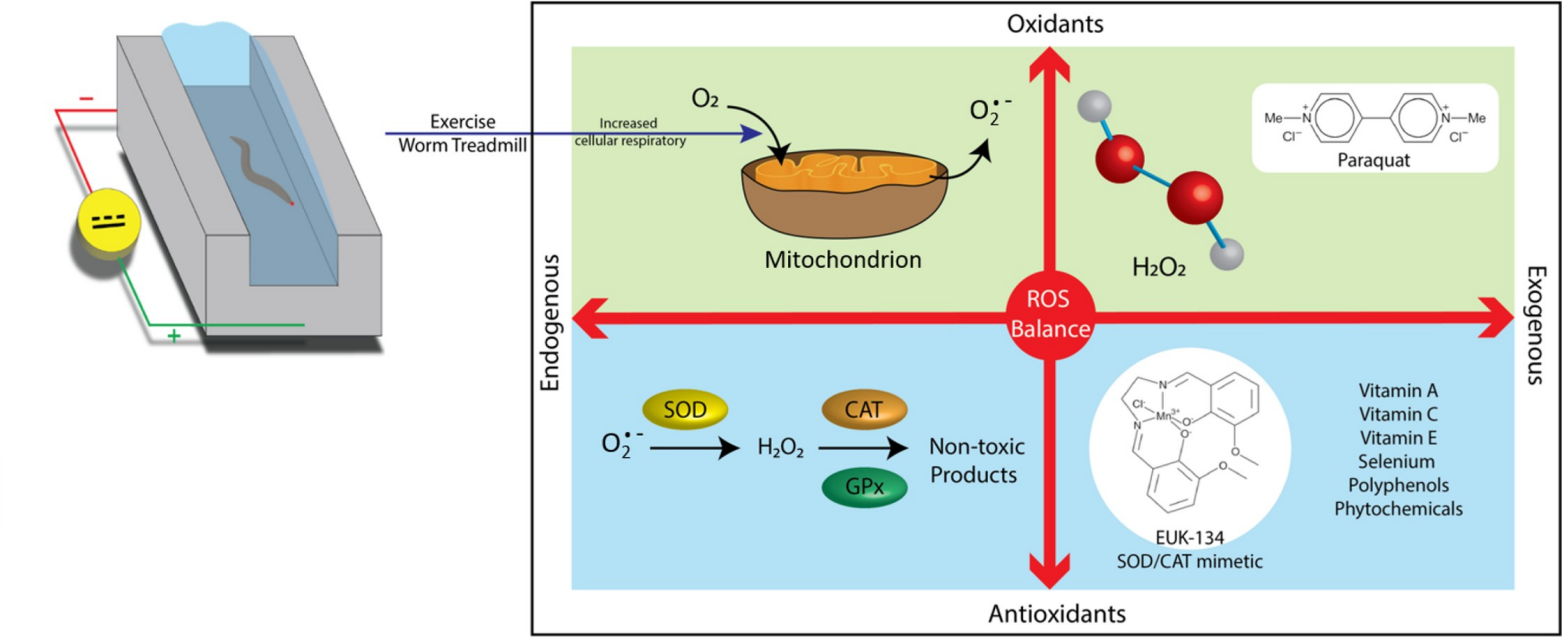
Relationships between oxidants and antioxidants.

The concept of this study is illustrated in Fig 1. We investigated the benefits of EUK-134 and some crucial endogenous antioxidant enzymes obtained from exercise through comparisons of aging markers between wild-type (N2) and SOD- and CAT-deficient (GA480 and LB90 (*ctl-2; him-8*), respectively) *C*. *elegans*. Changes in physiology, including lifespan, progeny, and kinetic power, were analyzed to evaluate the net impact of the treatments on the aging process of the nematode. Results indicated that the treatments successfully compensated for the adverse consequences of the absence of SOD and CAT. Observations also revealed that SOD insufficiency has a stronger negative influence on the worms than CAT deficiency. Finally, N2 was treated separately with the different treatment options under H_2_O_2_-induced stress conditions to assess its resistance to external stress and clarify the relationship between the environment, physiological status, and enzyme regulation.

## Materials and methods

### Worm treadmill system: Experimental setup and operation

A microchip was used to force the *C*. *elegans* to exercise via their electrotactic nature. The configuration of the chip was designed using the free software Dr. Engrave and manufactured by a computer-connected engraving machine (Computer Numerical Control, Roland, EGX-400). The chip consists of eight straight microchannels measuring 12 mm in length and 1 mm in width. Two electric wires were inserted and fixed at the end of the microchannels, thus allowing a DC electric field to be applied to the worms. The wire was covered in agar to prevent contamination of the buffer with impurities generated from the electrolytic reaction. The microchannels were filled with nematode growth medium (NGM) solution. Worms were trained daily for 10 min starting from the L4 stage. Ten minutes of exercise was implemented on the basis of our previous study [27]. The applied voltage ranged from 2 V_pp_ to 5 V_pp_, and the electrodes were inverted every 30 s to keep the worms swimming inside the channels.

### *C*. *elegans* culture

Bristol N2 was used as the wild-type strain. The transgenic strains LB90 *[ctl-2(ua90) II; him-8(e1489) IV]* and GA480 *[sod-2(gk257) I; sod-3(tm760) X]* were respectively used as the CAT- and SOD-deficient models. LB90 (*ctl-2; him-8*) nematodes demonstrate a shortened lifespan, whereas GA480 (*sod-2; sod-3*) nematodes show a slow growth rate, reduced brood size, and hypersensitivity to oxidative stress. Another strain, MAH99 (*muIs84 [sod-3p*::*gfp]*), was used for SOD-3 assay. The GFP gene was used as a reporter of *sod-3* gene expression in the transgenic strain. The worms were maintained at 20°C in an incubator and bred on agar dishes seeded with *Escherichia coli* strain OP50-1 as a food source according to standard protocols. All transgenic strains were obtained from the Caenorhabditis Genetics Center (CGC). All worms in the control group received no treatments but were cultured and handled the same way as other treated worms.

### Antioxidant supplementation

EUK-134 is a dark brownish-black powder (Bio Vision, US). The synthesized salen-manganese complex possesses stable low-molecular weight compounds and contains tightly bound manganese, which exhibits strong SOD and CAT activity. EUK-134 displays excellent pharmacological efficacy and efficiency in several oxidative stress-related disease models. In the current study, 199.2 mM EUK-134 was dissolved in DMSO, diluted into NGM medium as a 1 mM stock solution, aliquoted into 1.5 ml microcentrifuge tubes, and stored in a −20°C refrigerator to protect it from air and moisture. In the current study, 199.2 mM EUK-134 was initially obtained after dissolving in DMSO, diluted into NGM medium as a 1 mM stock solution, aliquoted into 1.5 ml microcentrifuge tubes, and stored in a −20°C refrigerator to protect it from air and moisture. The final weight ratio of DMSO in the solution was therefore only 0.5%, which greatly reduced the chance of nematode injury. Past literature [36, 37] showed DMSO weight ratio less than 1% to be quite safe for worms and make nearly no significant changes in worm’s physiological phenotypes. Nematodes were then treated with EUK-134 (1 mM) for 3 h in the study.

### Lifespan assay

Life span assay was conducted according to our previous study [26]. After age synchronization, L4 nematodes were divided into groups based on their treatment with exercise and/or antioxidants and transferred to new agar plates. Survivability was checked every day until the expiration of all worms. No sign of pharyngeal pumping in response to mechanical stimulation was observed, which indicates death. The worms that died on the walls of the Petri dishes were excluded. Data from lifespan assay were used to calculate the survival curve using Graphpad Prism 5.0 (Graphpad Software, La Jolla Inc.). Statistical significance between curves was analyzed by the Mantel–Cox log-rank test. Differences were considered significant only when the *p*-value was less than 0.05.

### Progeny assay

Progeny assay was performed as described in our previous publication [26]. Similar to the lifespan assay, the worms were divided, exercised, and/or administered antioxidants daily from L4 to adult day 7. After shifting original worms to new agar plates, the number of eggs and larvae on the old dishes was recorded to calculate the mean number of progeny produced each day (number of offspring per adult worm).

### Motility assay and derivation of kinetic power

Kinetic power, which is derived from the velocity fields of the movement of *C*. *elegans*, was used to evaluate the motility of the worms (S1 Fig). The detailed information of the working principle can be referred to our past literature [27]. A common flow visualization technique to measure velocity in microfluidics, micro particle image velocimetry (μPIV), was used to yield the velocity fields of the swimming worms. When restricting a worm in a mixed droplet of NGM buffer and polystyrene (PS) particles, the movement of the worm displaces the surrounding PS particles. A series of images recording the continuous motion of the animal is then separated into numerous interrogation windows and analyzed pair by pair to obtain each particle’s velocity and direction via the spatial cross-correlation algorithm; thus, an acceleration field can be derived, and the total force of the fluid, which is equivalent to the force exerted by the worm, can be calculated. The total energy of the worm can be calculated from the following equation, where $\overset{⃑}{F}$ is the total force and $\overset{⃑}{S}$ is the interrogation window displacement:

$$E=\overset{⃑}{F}∙\overset{⃑}{S}$$ (1)

Finally, the total energy is divided by the number of swimming cycles, time interval, and length of the nematode to obtain the unit kinetic power exerted by the worm.

A single animal was confined to a 0.63 μl NGM buffer droplet equally distributed with 4% PS tracer particles (*d*_p_ = 3.2 μm) (Thermo Fisher Scientific, USA) and sandwiched between two glass slides separated by a 110 μm strip of adhesive tape as a spacer. The whole droplet was placed in the field of view of an epifluorescence microscope (IX71, Olympus) under a 10× objective lens and a set of filters corresponding to the PS particles (Ex/Em: 540 nm/611 nm). A high-speed camera (GX3, NAC) captured a series of consecutive images of the swimming worm at a frame rate of 50 Hz. PS particle displacement was processed via the spatial cross-correlation algorithm using the freeware JPIV to determine the worms’ acceleration, which is then analyzed to calculate their kinetic power by using MATLAB.

### Exercise training and antioxidants treatment timeline

*C*. *elegans* was divided into eight groups: CON, three continuous-treatment groups (EXER, EUK, and EE), and three suspended-treatment groups (sEXER, sEUK, and sEE). Age-synchronization assay was performed before every experiment. Worms were treated in groups starting from the L4 stage. Details of the group treatments in each assay are provided in (S2 Fig).

### SOD-3 assay

The expression of *sod-3*::*gfp* in strain MAH99 (*sod-3p*) was monitored from adult day 1 to adult day 10 under a fluorescence microscope (IX71, Olympus) equipped with a GFP filter, a filter cube (450–490 nm excitation, 505 nm dichroic mirror, 530 nm barrier), and a 10× objective lens. Fluorescence images were captured by using a CCD camera (DP72, Olympus). Green image slices were extracted based on the relevant color channels and then analyzed by using the freeware ImageJ. Fluorescence intensities were defined by the mean pixel intensity of the selected region of the worm in green-level images. The whole animal body was selected for the fluorescent intensity in every animal.

### DHE assay

The ROS-sensitive dye DHE was supplied as a 5 mM solution in DMSO. An oxidized form of DHE, ethidium, was used to estimate ROS levels in the bodies of tested worms from adult day 1 to adult day 10. Following each group’s treatment, the worms were washed thrice with M9 buffer and incubated in DHE (diluted to 3 μM in M9 buffer) for 30 min at 30°C. Red fluorescence was detected and captured by an Olympus IX71 inverted microscope system with a 10× objective lens and a DP72 CCD camera. Fluorescence intensities of ROS (AU) were defined by the mean pixel intensity of the selected region of the worm in red-level images using ImageJ. The whole animal body was selected for the fluorescent intensity in every animal.

### Statistical analysis

All experimental data were assessed by Prism 7.0 (Graphpad Software, La Jolla Inc.) and are presented in the figures as mean ± standard deviation. One-way analysis of variance (ANOVA) was performed on all adult days in the motility assay, adult day 1 and adult day 2 in the short-term SOD-3 assay, long-term SOD-3 assay, and DHE assay. Two-way ANOVA was performed on adult day4 in the short-term SOD-3 assay or adult day 6 in the long-term SOD-3 assay and DHE assay to reveal differences between the treated and stopped treating groups. The other days were not examined herein because considering worms experienced homeostatic rebalance on adult day 3 and surge of aging stress on adult day 10. In all the statistical analyses, a *p*-value ≤ 0.05 was considered significant at a 95% confidence level.

## Results and discussions

### Movement and electrotaxis of *C*. *elegans* in the flow chamber

**Fig 2:**
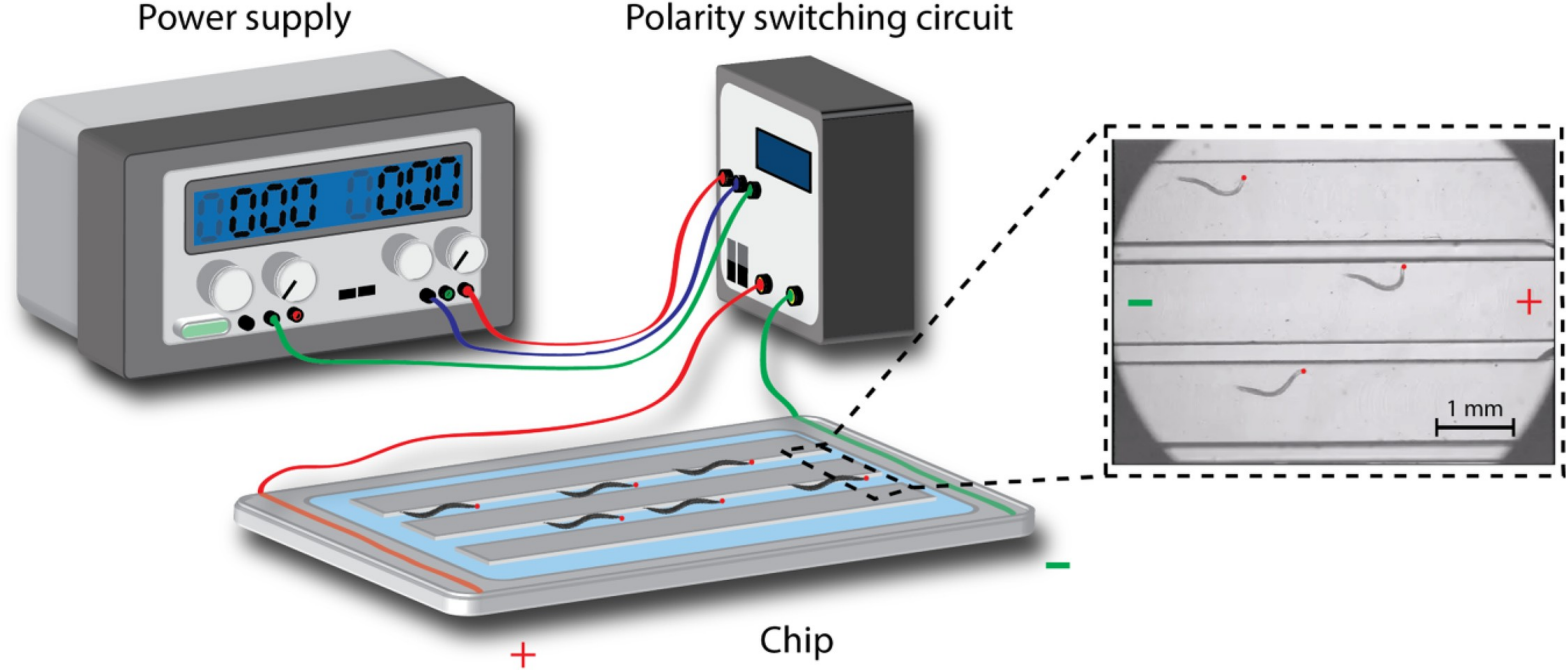
Schematic diagram of the electrotactic microchip setup used to impose exercise training on *C*. *elegans*. The flow chamber is connected to a DC power supply and a polarity-switching circuit. Worms are attracted to swim toward the negative pole by electrotaxis. Red dots indicate the head of the nematodes.

*C*. *elegans* is an amphibious animal that tends to move more vigorously in a liquid environment than on an agar plate. However, worms in liquid swim in a rather spontaneous and unpredictable pattern. To induce controllable behavior in the worms, we employed their electrotactic nature to guide them to swim consistently within a confined flow chamber. The electrotactic property of *C*. *elegans* is a neuronal response to an electrical stimulus in which the nematodes show attraction to a negatively charged pole [38]. This behavior is highly reproducible, direction sensitive, and voltage dependent. We fabricated a flow chamber system consisting of a voltage generator, polarity switching circuit, microchip with worm runways, and cover glass to impose exercise treatment on the worms (Fig 2). The orientation of worm movement was periodically reversed by switching the electrical polarity of the circuit to confine the animals to swim inside the microchannels. Previous studies have reported that worms tend to respond to electrical stimuli depending on their developmental stage [38]. Our observations showed that early larval worms express ambivalent swimming orientations when exposed to an electric field because of their underdeveloped electrosensory neurons [13]. Therefore, all experiments in this study were conducted on L4 worms. Paralytic phenotypes, such as body curling and trembling, appeared when the applied voltage exceeded the worms’ electrical sensing capacity. To avoid injury to the worms, the electrical intensity was kept between 2 and 5 V_pp_, thus ensuring a field intensity below 2.5 V_pp_/cm [26, 27]. The voltage applied was initially set to 5 V_pp_ for L4 worms but progressively decreased to 2 V_pp_ as the developmental stage of the animals increased.

### Physiological phenotypes of N2 and CAT/SOD-deficient *C*. *elegans* under various treatments

Three worm strains, including wild-type N2, CAT-deficient LB90 (*ctl-2; him-8*), and SOD-deficient GA480 (*sod-2; sod-3*), were used in this study to evaluate the effects of antioxidative stress resulting from exercise training and antioxidant supplementation (S1 Video: N2, S2 Video: LB90 (*ctl-2; him-8*), and S3 Video: GA480 (*sod-2; sod-3*)). The supplementary videos were taken in the early adult stage of all worms. Unlike humans, *C*. *elegans* has five *sod* genes, where *sod-1*, *sod-2*, and *sod-4* are the primary cytoplasmic, mitochondrial, and extracellular *sod* genes, respectively; whereas *sod-3* and *sod-5* are inducible mitochondrial and cytoplasmic *sod* genes, respectively. Considering exercise upregulates mitochondrial activity and stress response, both *sod-2* and *sod-3* seem to be ideal biomarkers. As compared with the ubiquitous expression of *sod-3*, however, *sod-2* mainly expressed in the head and tail regions. As a result, *sod-3* was more preferably selected for ROS monitoring in the study. The physiological phenotypes of the worms were examined using lifespan and progeny as key indicators. Under normal conditions (i.e., without stimulation or treatment), N2 worms exhibited longer lifespans and more progeny than their LB90 (*ctl-2; him-8*) and GA480 (*sod-2; sod-3*) counterparts. Each strain was divided into four groups, including the exercise (EXER) group, the EUK-134 supplementation (EUK) group, the combined exercise and antioxidant supplementation (EE) group, and the control (CON) group, and each group was prescribed a different treatment to facilitate our investigation. The results of survival analysis and progeny count of all strains showed that EE yields the best benefits, followed by EXER, EUK, and CON.

**Fig 3:**
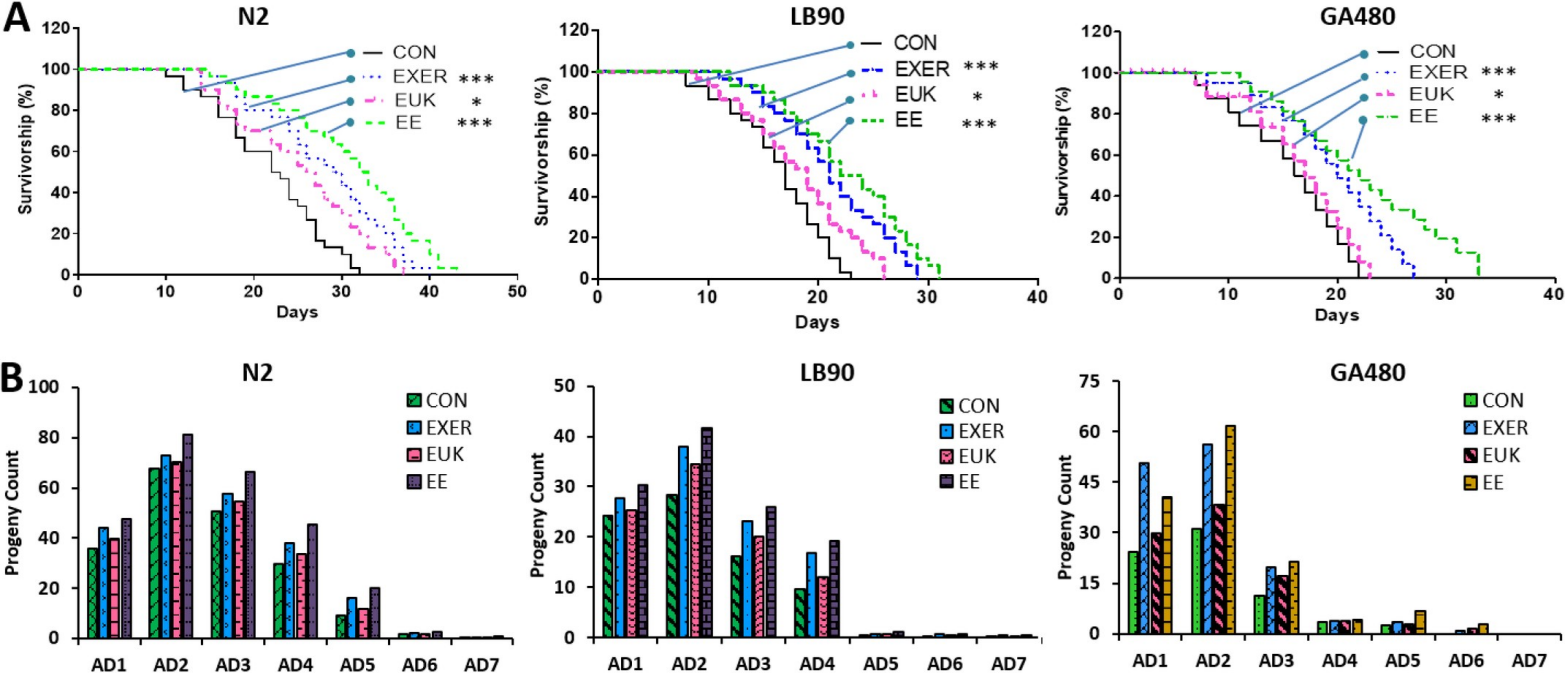
Physiological phenotypes of each strain under different treatments. (A) Lifespans of N2, LB90 (*ctl-2; him-8*), and GA480 (*sod-2; sod-3*). The mortality data were subjected to log-rank (Mantel–Cox) test survival analysis to prepare survival curves and compared with those of untreated worms (*n* = 30). The symbols *, **, and *** denote *p* < 0.05, *p* < 0.01, and *p* < 0.001, respectively, compared with the control. (B) Average progeny counts of N2, LB90 (*ctl-2; him-8*), and GA480 (*sod-2; sod-3*) during the adult stage from adult day 1 (AD1) to adult day 7 (AD7) (*n* = 30).

The lifespan of the worms was significantly extended by the treatments (Fig 3A). Lifespan and progeny assays were conducted following the timelines detailed in (S2A and S2B Fig). Our previous research confirmed that EXER-treated worms can live longer than their control counterparts [13]. In the current study, EUK-134 revealed supportive effects not only on the EUK group but also in the EE group. In N2 worms, the median survival lifespans of the EE, EXER, and EUK groups were 32.5, 29.5, and 26.5 days, respectively; these lifespans are longer than that of the corresponding CON group (22.5 days). Untreated LB90 (*ctl-2; him-8*) and GA480 (*sod-2; sod-3*) worms showed shortened lifespans compared with normal N2 worms, which means the lack of active enzymes working in the oxidative stress pathway leads to excess ROS accumulation in the two former strains. Nonetheless, the median lifespans of LB90 (*ctl-2; him-8*) and GA480 (*sod-2; sod-3*) worms showed significant extensions after treatment. Among the four experimental groups, the EE groups of the LB90 (*ctl-2; him-8*) and GA480 (*sod-2; sod-3*) strains exhibited maximum improvements of up to 35% and 33%, respectively. The median survival of the LB90 (*ctl-2; him-8*) strain was slightly longer than that of the GA480 (*sod-2; sod-3*) strain. This result implies the pivotal role of the upstream genes *sod2* and *sod3* in regulating antioxidant enzyme activity. Knockdown of these genes, especially those upstream ones, tends to result in abnormal ROS accumulation. Thus, elevated oxidative stress may cause more severe damage (low survivorship and low progeny count) to GA480 (*sod-2; sod-3*) worms than to LB90 (*ctl-2; him-8*) worms, and the former may be more vulnerable to environmental stresses than the latter. We reasoned that the *cat-2* depleted worms (LB90 (*ctl-2; him-8*)) were less susceptible to damage as compared to the *sod-2/sod-3* depleted worms (GA480 (*sod-2; sod-3*)) because there are several other enzymes that scavenge H_2_O_2_ besides catalase, but there are no other enzymes that scavenge superoxide except for SOD.

Data from the progeny assay illustrated a trend similar to the results of survivorship assessment. When no treatment was applied, N2 worms showed the highest progeny count (progeny count = 194), followed by LB90 (*ctl-2; him-8*) worms (progeny count = 80) and then GA480 (*sod-2; sod-3*) worms (progeny count = 73) (S3 Fig). All treatment groups (EXER, EUK, and EE) of the three tested strains show improved fertility compared with the corresponding CON group. Moreover, EE-treated worms tended to lay more eggs, followed by EXER- and then EUK-treated worms (S3 Fig). Although the fertility of all treated strains improved as compared with their controls, gene-deficient worms still laid fewer eggs than wild-type worms. The progeny count of LB90 (*ctl-2; him-8*) worms markedly decreased on adult day 5, whereas the progeny count of GA480 (*sod-2; sod-3*) worms decreased even earlier on adult day 3 (Fig 3B). These outcomes explicitly indicate that the treatments could only partially restore the fertility of worms, not completely reverse the negative impact of genetic defects. The literature states that *ctl-2* deficiency may account for the shortened lifespan and reduced egg-laying capacity of nematodes [39]. Therefore, a decrease in brood size is likely a consequence of excess ROS accumulation [40]. Previous research has also demonstrated that *sod-2* is involved in sperm activation in *C*. *elegans* [41], and abnormalities in the reproductive functions of GA480 (*sod-2; sod-3*) may account for its observed reduction in progeny count. In our past studies, mitochondria in EXER-treated worms tended to be large and abundant, and muscle fibers were tightly maintained after training. Therefore, increases in progeny can be interpreted as a consequence of improvements in muscular strength (body wall muscle cells), mitochondrial function (fused mitochondria in the body wall muscle cells), and low ROS, all of which render the worms healthier. Our investigations of lifespan and progeny count suggest that combined treatment, rather than exercise or antioxidant supplementation alone, could boost the physiological performance or overcome the genetic defects of *C*. *elegans*.

### Motility assay of treated-wild type and gene-deficient *C*. *elegans*

**Fig 4:**
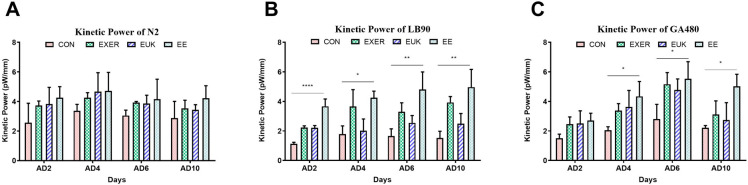
Long-term motility changes in strains (A) N2, (B) LB90 (*ctl-2; him-8*), and (C) GA480 (*sod-2; sod-3*) under different treatments. Motility was expressed in terms of kinetic power with labelled p-values based on one-way ANOVA. The symbols *, **, and **** denote *p* < 0.05, *p* < 0.01, and *p* < 0.0001, respectively, as compared with the control. The error bars represent standard deviations.

Motility assay was conducted in the present study following the same timeline of the lifespan assay (S2A Fig) to understand the regulation of worm power by exercise and antioxidant supplementation. Motility change was expressed in terms of kinetic power. While treatments in wild type strain N2 showed no significant differences, all treatment groups (EXER, EUK, and EE) of the two mutant strains (LB90 (*ctl-2; him-8*), and GA480 (*sod-2; sod-3*)) revealed the changes in motility compared with their respective controls over the observation period (from adult day 2 to adult day 10) according to one-way ANOVA test, thus suggesting that the prescribed treatments elevated body wall muscular strength in both wild-type and gene-deficient worms (Fig 4). Based on our prior research, the nematodes are considered to be in their prime from adult day 2 to adult day 6; hence, adult day 6 was considered the beginning of their middle-age period [13]. Gene-deficient worms receiving treatments expressed higher kinetic power than their control counterparts. This tendency implies that some complementary signaling pathways in CAT- and SOD-deficient animals are upregulated to promote other antioxidant-coding genes, detoxify proteins or biochemical, and provide an extra motility boost. The experiments were conducted until adult day 10 to reveal the extended effect of the treatments and motility changes with age. The kinetic power resulting from EUK-134 treatment was the lowest in LB90 (*ctl-2; him-8*). This difference can be likely attributed to the SOD mimetic property of EUK-134. Therefore, the EUK group in LB90 (*ctl-2; him-8*) worms could not improve their kinetic power as good as those in N2 and GA480 worms after taking up EUK-134. In addition, the kinetic power of GA480 (*sod-2; sod-3*) worms declined immediately after adult day 6, faster than that observed in LB90 (*ctl-2; him-8*) worms. This outcome suggests that *sod-2*/*sod-3* deficiency may cause more severe damage than *ctl-2* deficiency and lower the worms’ capacity to deal with oxidative stress.

### Effects of short-term treatment on *sod-3* expression

**Fig 5:**
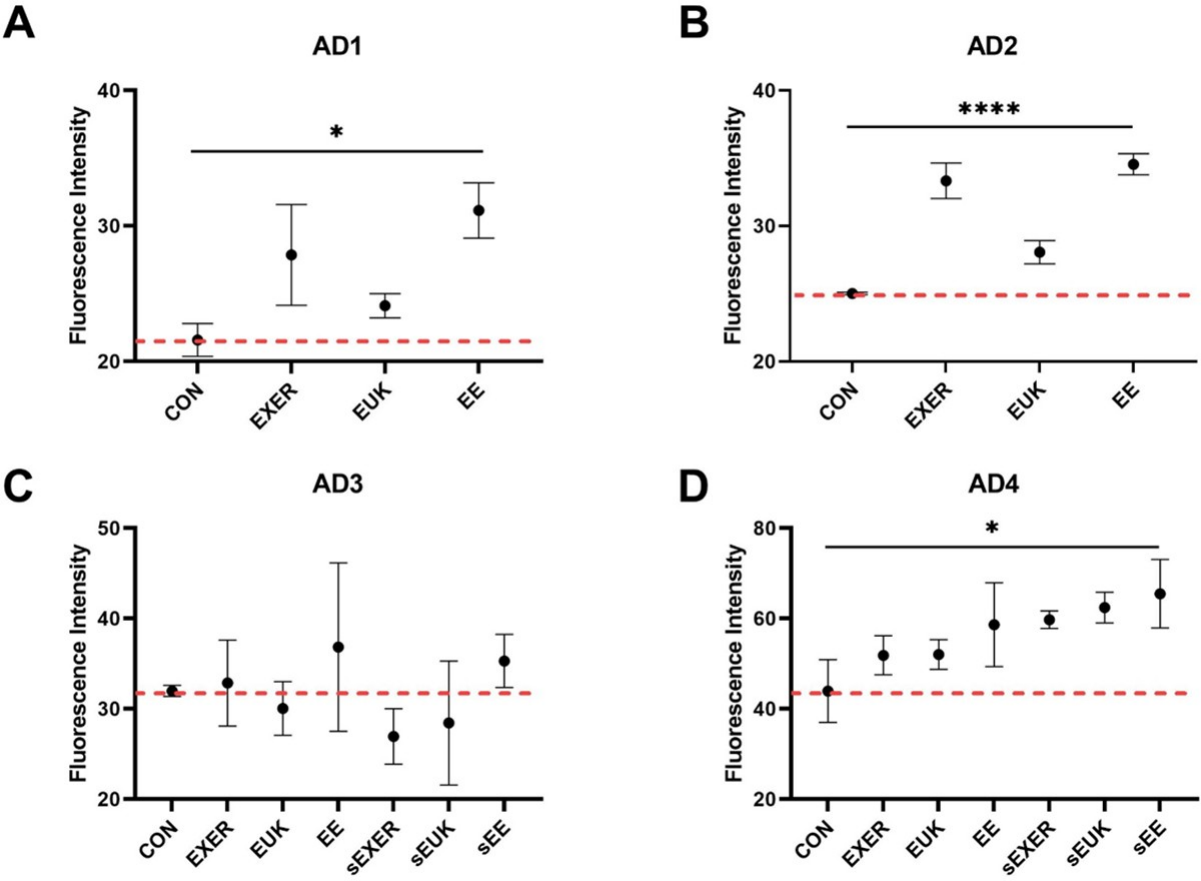
Short-term assessment of *sod-3*::*gfp* expression in MAH99 (*sod-3p*) under different treatments through fluorescence intensity measurements. (A) Adult day 1 (AD1); (B) Adult day 2 (AD2); (C) Adult day 3 (AD3); (D) Adult day 4 (AD4). Signals were measured 30 min after daily treatment for 4 days (*n* ≥ 3). The symbols *, and **** denote *p* < 0.05, and *p* < 0.0001, respectively, as compared with the control via one-way ANOVA (AD1 and AD2) and two-way ANOVA (AD3 and AD4) tests. The error bars represent standard deviations.

We conducted further assessments of biochemical indices, including SOD-3 levels, to discover the molecular mechanisms underlying the benefits of the treatments. *sod-3* is located in the mitochondrial matrix in *C*. *elegans* [42–44] and considered an indicator of intracellular antioxidants induced by stressful events; thus, *sod-3*::*gfp* fluorescence signals can represent changes due to different treatments at a specific time point using the transgenic *C*. *elegans* strain MAH99 (*sod-3p*). MAH99 (*sod-3p*) is a transgenic strain that green fluorescent protein (GFP) is used as a reporter of *sod-3* gene expression (S4 Video). Therefore, MAH99 (*sod-3p*) was used herein in the short-term and long-term SOD-3 assays. Detailed timeline conditions in different groups can be referred to in (S2C Fig). The short-term response of the worms was measured 30 min after exercise training and antioxidant supplementation. Given that *sod-3* is induced in a stressful environment, exercise-induced mild oxidative stress caused remarkable increases in SOD-3 levels in the EXER and EE groups as compared with the CON group on adult day 1 and adult day 2 (Fig 5). One-way ANOVA analyses indicated that the treatment methods indeed caused significant differences in the worm’s SOD levels in the first phase (adult day 1 and adult day 2). In the second phase (adult day 3 and after), two-way ANOVA was performed on adult day 4. The overall result indicated that *sod-3*::*gfp* signal was enhanced with any types of treatments. By contrast, MAH99 (*sod-3p*) worms that received sole EUK-134 supplementation also showed mild elevations of SOD-3 transcription on adult days 1, 2, and 4, but their SOD-3 levels were relatively lower than other treatment groups (nearly equal to the EXER group on adult day 4). This trend implied that EUK-134 has inferior effect in upregulation of intracellular SOD-3 as compared with most other treatment groups. Notably, three additional groups, sEUK, sEXER, and sEE, in which the treatments were suspended were added to the investigation after adult day 2. In comparison to the groups receiving continual treatments (EUK, EXER, and EE), the treatment-suspended worms appeared to continually present high *sod-3* expression on adult day 4 despite a short homeostatic rebalance on adult day 3. The benefits of exercise training or antioxidant supplementation may be extended. Fluorescence intensities relative to the CON group on adult day 3 revealed SOD-3 levels in all groups but seemed to have more impact on the sEXER, sEUK, and sEE groups than in the EXER, EUK, and EE groups. However, the short-term treatments remained to yield oxidative stress resistance, as SOD-3 levels in all treatment groups have a significant jump the following day, thus conjecturing the occurrence of a biochemical event on adult day 3 triggering a subsequent boost in *sod-3* production to protect the worms. The possible biochemical events may include accumulated ROS after reproduction [45, 46] and a sudden surge of stress induced by the suspended treatments. Similar to the groups receiving treatments, the sEE group showed the highest SOD-3 level among the groups that stopped receiving treatments on adult day 4.

### Effects of long-term treatment on *sod-3* expression

**Fig 6:**
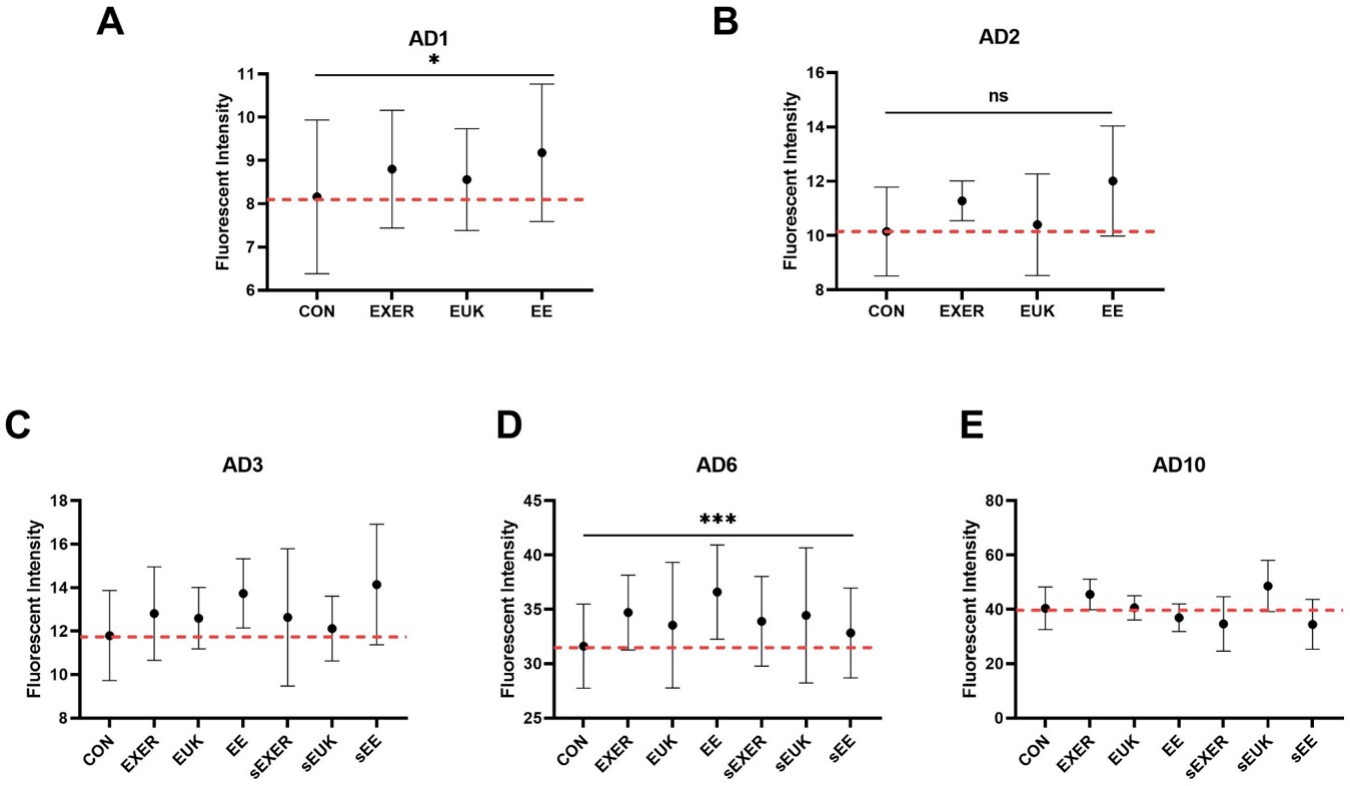
Modified SOD-3 assay clarifying the relationship between treatments, external stress, and aging stress. (A) Adult day 1 (AD1); (B) Adult day 2 (AD2); (C) Adult day 3 (AD3); (D) Adult day 6 (AD6); (E) Adult day 10 (AD10). The first phase of SOD-3 assay with the treatments was suspended on adult day 2 in the sEXER, sEUK, and sEE groups. Fluorescence intensity was measured on adult day 1, adult day 2, adult day 3, adult day 6, and adult day 10 (*n* ≥ 10). The symbols * and *** denotes *p* < 0.05 and *p* < 0.001, respectively, as compared with the control via one-way (AD1 and AD2) and two-way (AD6) ANOVA tests. The error bars represent standard deviations.

To investigate the roles of long-term endogenous stresses (e.g., aging, exercise) on *sod-3* expression in *C*. *elegans*, we implemented some modifications to the previous short-term SOD-3 assay. In the modified SOD-3 assay, the treatments were suspended in the sEUK, sEXER, and sEE groups after adult day 2, and the entire assay was completed on adult day 10 (S2D Fig). Fluorescence intensity from *sod-3*::*gfp* was measured 180 min after the treatments were completed for each group. The results on adult day 6, which is the start point of aging, showed that the exercise-treated groups (EXER and EE) significantly improved their resistance to external oxidative stress by upregulating SOD-3 levels and that treatment with EUK-134 (EUK) alone induced only slightly noticeable changes in SOD-3 signals (Fig 6). Similar to the previous short-term assessment, the benefits of treatments were extended (from adult day 3 to adult day 6) in all treatment groups but mitigated in the late adult stage (adult day 10) when compared with the corresponding controls. Similar to the short-term SOD-3 assay, one-way ANOVA analyses also indicated that the treatment methods cause significant differences in the worm’s SOD levels on adult day 1. In the second phase (adult day 3 and after), two-way ANOVA was performed on adult day 6. Although the SOD-3 levels revealed no drastic differences between the continually-treated and treatment-suspended groups, all those groups still showed comparable SOD-3 levels with their respective controls, indicating that *C*. *elegans* can benefit from antioxidant supplements and training as long as they receive treatments. The potent antioxidant properties of the treatments induced young physiological phenotypes, such as strong motility, long lifespan, and high fertility, in older worms. Unlike the finding in the previous short-term SOD-3 assay, however, the ability of the worms to produce SOD-3 appeared to decrease as they grew to later adult stages (adult day 10). Late-stage endogenous stress produced by aging appears to overwhelm the benefits of the treatments, leading to oxidative damage resulting from ROS overexpression in the worm body.

### DHE assay for ROS levels in treated worms

**Fig 7:**
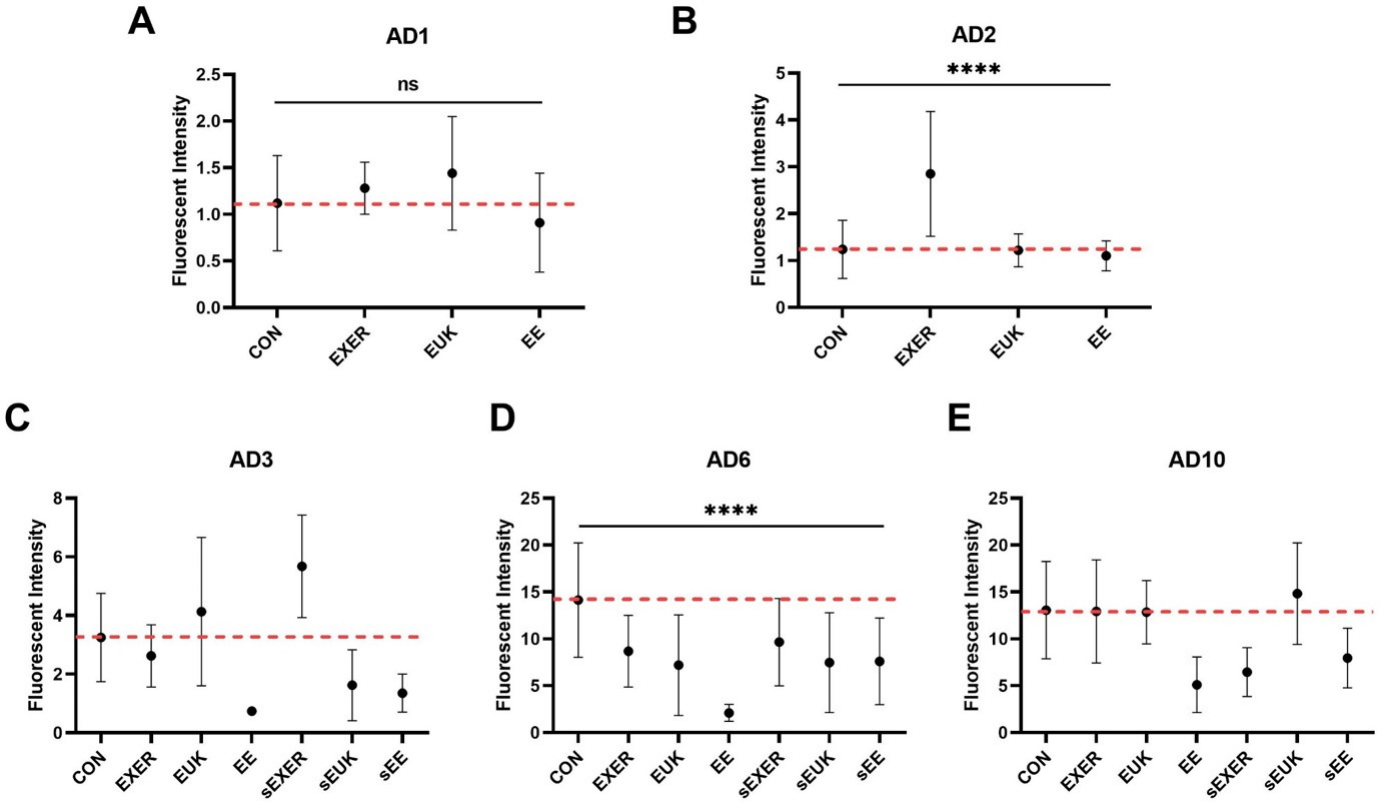
Long-term superoxide detection using the DHE assay. (A) Adult day 1 (AD1); (B) Adult day 2 (AD2); (C) Adult day 3 (AD3); (D) Adult day 6 (AD6); (E) Adult day 10 (AD10). Treatments in the sEXER, sEUK, and sEE groups were suspended on adult day 2. Fluorescence intensity of the wild-type N2 strain under different treatments was measured on adult day 1, adult day 2, adult day 3, adult day 6, and adult day 10(*n* ≥ 10). The symbol **** denotes *p* < 0.0001 as compared with the control via one-way (AD1 and AD2) and two-way (AD6) ANOVA tests. The error bars represent standard deviations.

Dihydroethidium (DHE) is a fluorescent dye used to monitor the overall ROS level. After free permeation through the cell membrane, DHE can interact with cellular superoxide to generate a highly specific red fluorescent product, 2-hydroxy ethidium (2-OH-E(+)). Under the same experimental scheme (S2D Fig), the EE, sEUK, and sEE groups maintained lower ROS levels from adult day 3 to adult day 10 when compared with the CON group. Whereas, other treatment groups (i.e., EXER, sEXER, and EUK) showed slight fluctuations in ROS levels on adult day 3 and low ROS levels on adult day 6 but the same ROS levels as the control on adult day 10 (Fig 7). Notably, the EE, sEUK, EXER, and sEXER groups appeared to maintain low ROS levels for a short while as compared with the CON group, thus implying that they may possess some antioxidative capability. Similar to findings in the previous long-term SOD-3 assay (Fig 6), groups with suspended treatments (i.e., sEXER, sEUK, and sEE) also showed resistance to endogenous ROS attack from adult 3 to adult day 6 Among the suspended treatment groups, the sEXER and sEE groups showed a longer extended effect than the sEUK group. This benefit, however, appeared to decrease as the worms grew to the late adult stage (adult day 10). The trend observed became especially remarkable after the worms stopped laying eggs. We thus suppose that senescence may be a factor compromising the worms’ ability to respond to the treatments, hence weakening their overall health and ROS homeostasis. Overall, one-way ANOVA analyses indicated that the treatment methods cause significant differences in the worm’s ROS levels in the entire measurement period except adult day 1. While we looked into the ROS levels between continually-treated and treatment-suspended groups on adult day 6, no different effects were observed according to two-way ANOVA. Despite the uncertain differences due to large standard deviations, a clear trend was low mean ROS levels in all groups on adult day 6.

## Conclusion

Exercise is well known to be an effective measure to avoid senescence and rejuvenate body functions. Numerous studies on exercise have been conducted on higher animals, but none have involved simple animals, such as *C*. *elegans*. In the current study, we implemented a microfluidic device to assist the exercise training of *C*. *elegans* through electrotaxis. An oxidative stress model was hypothesized to correlate ROS relief and exercise. For comparison, the synthetic SOD/CAT-mimetic EUK-134, an exogenous antioxidant, was used to investigate oxidative stress relief in the worms. Our findings confirmed that all treatments impart beneficial effects and boost physiological phenotypes, including extended lifespan and enhanced reproductive capacity. However, the improvements of kinetic power and SOD-3 levels seemed to be compromised by increased ROS levels when worms entered late adult stage (adult day 10). Genetic defects in cellular antioxidant enzymes, such as SOD and CAT, have been reported to induce adverse effects in worms [39, 47]. The benefits of the endogenous and exogenous treatments appeared to compensate for these genetic defects by upregulating other antioxidant pathways [48, 49].For example, the rise of ROS production can promote activation of SKN-1 and DAF-16 by the p38 MAPK pathway In addition to the complementary ROS enzymes [50, 51]. Both SKN-1 and DAF-16 are inhibited under normal growth conditions in *C*. *elegans* by kinases in the insulin/insulin-like signaling pathway. Conversely, this inhibition will be lifted to allow the SKN-1 and DAF-16 to abundantly produced under abnormal ROS exposure to trigger a transcriptional program that induces several antioxidant and stress-protective enzymes [52, 53].

Kinetic power analysis proved that the effects of both *sod-2*/*sod-3* and *ctl-2* deficiencies are more severe than those of wild-type worms because the motility of LB90 (*ctl-2; him-8*) and GA480 (*sod-2; sod-3*) strains is more seriously impaired in the early adult stage compared with that of the N2 strain. However, each strain was influenced differently under treatments and attained various degrees of recovery. Treatments involving exercise training, such as EXER and EE, raised SOD-3 levels. However, the benefits brought about by the treatments seemed to be compromised by aging, especially in the late stage of adult worms. EUK-134 demonstrated weak ability to induce *sod-3* expression under normal conditions, thus suggesting that EUK-134 can only scavenge ROS, not coordinate with other antioxidant pathways. Nevertheless, ROS levels in EUK and sEUK groups were maintained at relatively low levels. The SOD-3 and DHE assays proved that all treatments are capable of providing beneficial effects by mitigating excess ROS. In addition, the beneficial effects observed lasted for several days even after cessation of treatments. The EE group notably showed the lowest ROS accumulation and the highest *sod-3* expression among the groups studied, indicating the best anti-oxidative capacity. This effect seems to be similar to that in the sEE group. In the present work, to the best of our knowledge, a combination of electrotaxis-based exercise and antioxidant treatment was successfully performed on *C*. *elegans* for the first time. We hope the findings and the worm exercise platform will inspire future researchers to seek other molecular biology mechanisms underlying oxidative stress relief resulting from exercise and external antioxidant supplementation.

## Supporting information

S1 Fig Step-by-step procedure for the derivation of kinetic power. (DOCX)

S2 Fig Timelines for (A) lifespan assay/motility assay, (B) progeny assay, (C) short-term SOD-3 assay, and (D) long-term SOD-3 assay/DHE assay.
(DOCX)

S3 Fig Progeny counts of N2, LB90 (*ctl-2; him-8*), and GA480 (*sod-2; sod-3*) strains in CON, EXER, EUK, and EE groups. (DOCX)

S1 Video Swim gait of a N2 young adult worm. The worm was confined in a sandwiched NGM droplet and recorded with a 4× objective under the white light mode. The height of the spacer was 110 μm.
(AVI)

S2 Video Swim gait of a LB90 (*ctl-2; him-8*) young adult worm. The worm was confined in a sandwiched NGM droplet and recorded with a 4× objective under the white light mode. The height of the spacer was 110 μm.
(AVI)

S3 Video Swim gait of a GA480 (*sod-2; sod-3*) young adult worm. The worm was confined in a sandwiched NGM droplet and recorded with a 4× objective under the white light mode. The height of the spacer was 110 μm.
(AVI)

S4 Video Swim gait of a MAH99 (*sod-3p*) young adult worm. The worm was confined in a sandwiched NGM droplet and recorded with a 4× objective under the white light mode. The height of the spacer was 110 μm.
(AVI)
