# Supplementary figures and images for: Effects of electrotactic exercise and antioxidant EUK-134 on oxidative stress relief in Caenorhabditis elegans
Source: PLoS One. 2021 Jan 20;16(1):e0245474. doi: 10.1371/journal.pone.0245474 (PMC7817057; doi:10.1371/journal.pone.0245474)

**
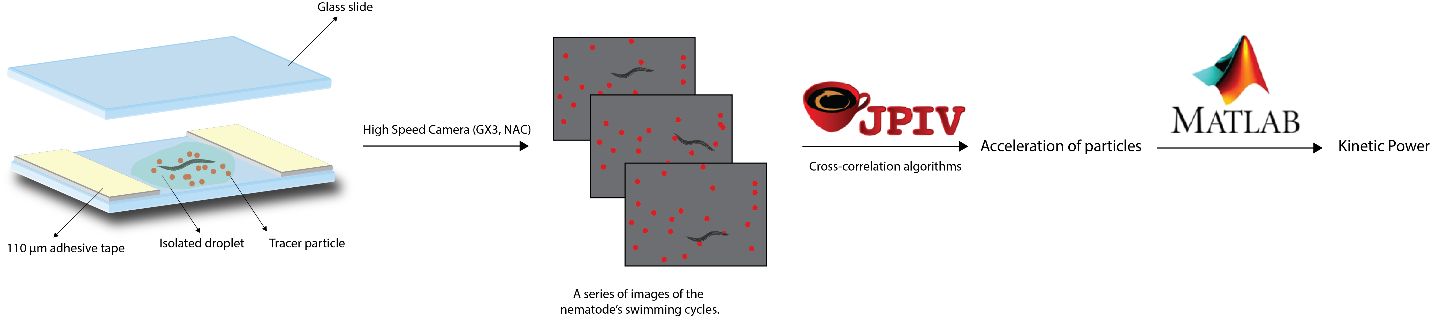
**

**S1 Fig.** Step-by-step procedure for the derivation of kinetic power.

Supplement: S1 Fig — (DOCX) [file pone.0245474.s001.docx]

**
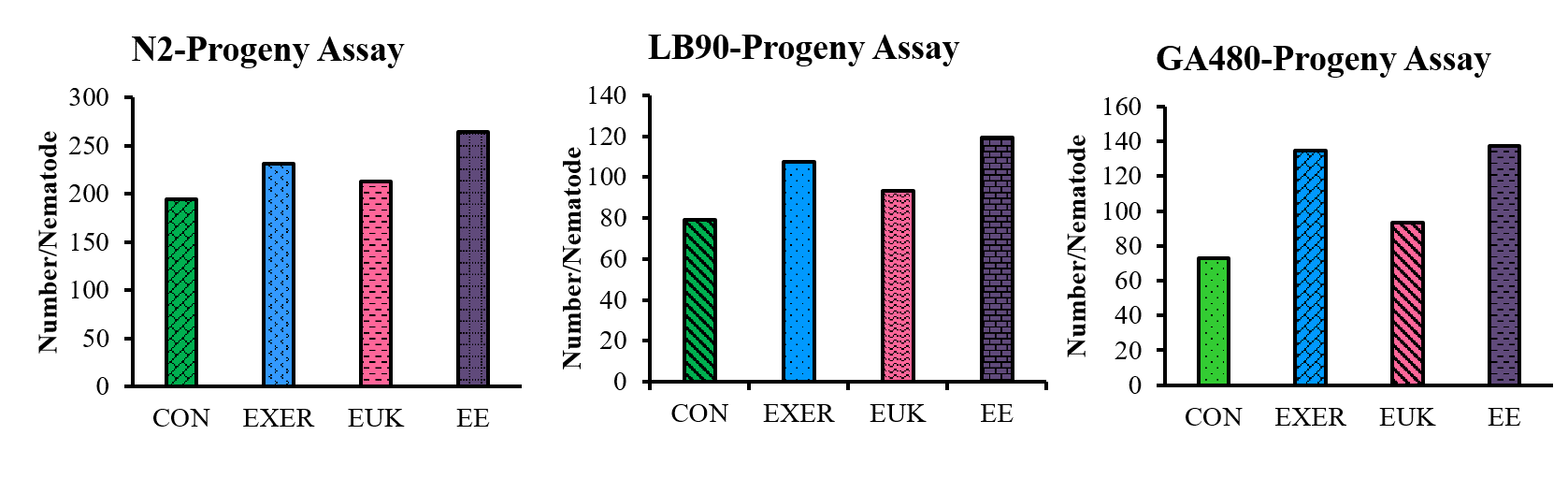
**

**S3 Fig.** Progeny counts of N2, LB90, and GA480 strains in CON, EXER, EUK, and EE groups.

Supplement: S3 Fig — (DOCX) [file pone.0245474.s003.docx]
