# Supplementary material for: Effects of electrotactic exercise and antioxidant EUK-134 on oxidative stress relief in Caenorhabditis elegans
Source: PLoS One. 2021 Jan 20;16(1):e0245474. doi: 10.1371/journal.pone.0245474 (PMC7817057; doi:10.1371/journal.pone.0245474)
Supplement: S2 Fig — Timelines for (A) lifespan assay/motility assay, (B) progeny assay, (C) short-term SOD-3 assay, and (D) long-term SOD-3 assay/DHE assay. (DOCX) [file pone.0245474.s002.docx]

**
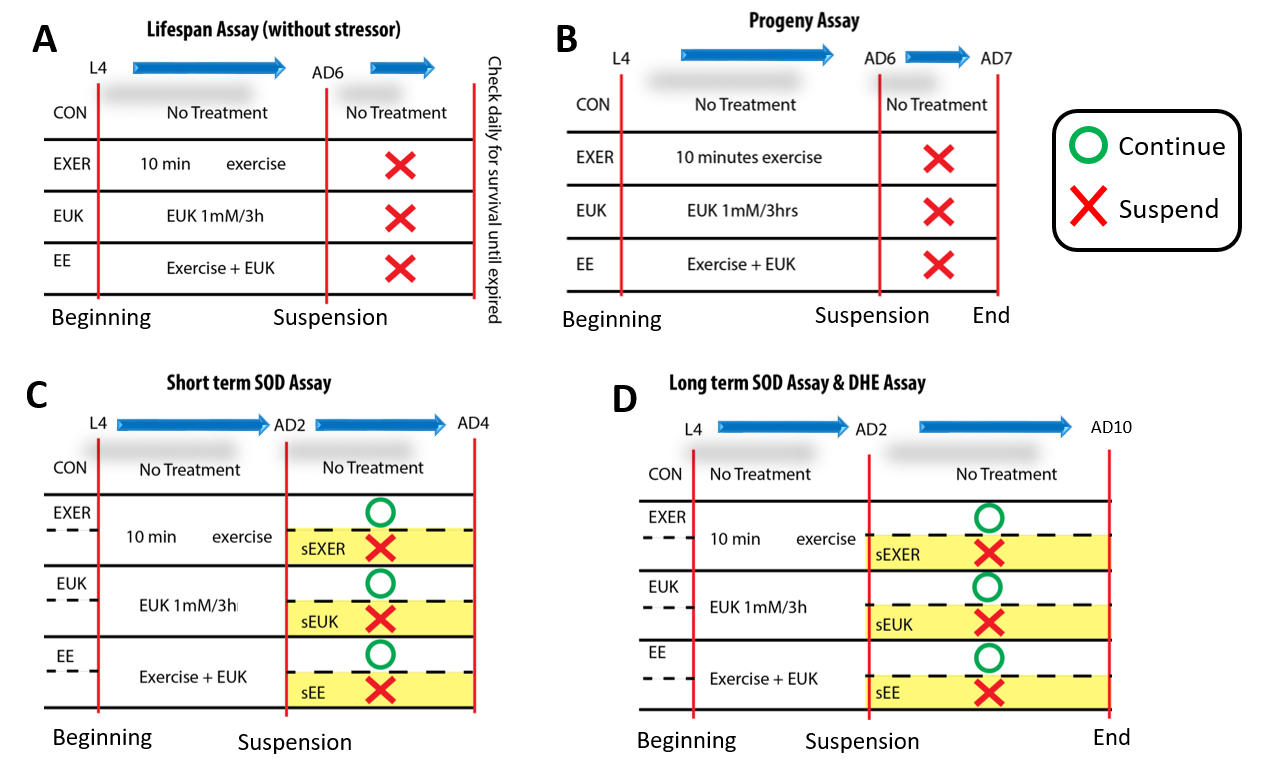
**

**S2 Fig.** Timelines for (A) lifespan assay/motility assay, (B) progeny assay, (C) short-term SOD assay, and (D) modified SOD assay/DHE assay.
